# Finite Volume Method Modeling of Heat Transfer in Acoustic Enclosure for Machinery

**DOI:** 10.3390/ma15041562

**Published:** 2022-02-19

**Authors:** Jakub Wróbel, Urszula Warzyńska

**Affiliations:** Faculty of Mechanical Engineering, Wrocław University of Science and Technology, Łukasiewicza 7/9, 50-371 Wrocław, Poland; urszula.warzynska@pwr.edu.pl

**Keywords:** acoustic enclosure, heat transfer, computational fluid dynamics, noise

## Abstract

This paper deals with the problem of heat accumulation in acoustic enclosures. Increased noise levels at production sites or manufacturing lines force the application of acoustic enclosures. Effective noise reduction due to enclosures often comes with the additional thermal insulation of the device, which in many cases causes a strong increase in the device operation temperature. This paper presents the methodology of thermal phenomena numerical modeling based on the potential influence of acoustic enclosures on the increase in device operation temperature. The proposed model consists of an original acoustic enclosure concept design, and the numerical modeling is based on the computational fluid dynamics FVM (finite volume method) conducted in Ansys Fluent. The research comprised a set of simulations at different air flow rates of 52.5 m^3^/h, 105 m^3^/h, 210 m^3^/h and 420 m^3^/h at the enclosure inlet. The analysis carried out on the basis of flow paths and temperature distribution plots inside the enclosure led to the conclusion that the expected, analytically calculated minimum volumetric flow rate is not sufficient to effectively cool the investigated device to the required temperature of 26 °C, and higher air flow rates should be applied. Simulation results indicated that the numerical tools can be useful in the prediction of the heat exchange process, as well as in the selection of an appropriate source and location of cooling.

## 1. Introduction

Noise and vibration harshness is an important and current problem of many industrial devices. Increased levels of equivalent sound pressure may impact the wellbeing and health of any individual present in the area of the noisy machine [1,2]. The distribution of personal protective equipment is the most common way to reduce the negative impact of noise on operators. Unfortunately, the problem is commonly spreading in larger areas, such as residential regions. The increase in the distance between the noise source and the point at which exposure occurs reduces the registered sound pressure levels; however, the reduction is often not sufficient to meet the allowed day or night noise values. Long-term exposure, even at sound levels that are not annoying in the case of brief exposures, can induce health problems, and reduce the quality of life and mental performance [3,4,5].

Targeting the noise at the source is one of the most effective ways to deal with the problem. Changes in the construction of the device itself or changes in drive speeds, machine positioning and types of support have been proven to be effective in the fight against noise. Large industrial production devices often include some application-specific design changes that incorporate noise reduction measures, including a special type of foundation, vibration isolation, production hall equipment distribution, and wall and ceiling acoustic proofing. The scope of this article is a group of small to mid-size machinery which is not usually equipped with noise-reducing means or included in the industrial site design plan.

Acoustic enclosures and barriers should be used as a last resort measure to reduce the noise generated during the operation of any kind of machinery. Unfortunately, in some cases, the use of acoustic enclosures cannot be avoided.

### 1.1. Acoustic Enclosures

Materials of various mechanical and acoustic properties are used for acoustic enclosures. Most commonly, the exterior of the enclosure consists of a mechanically robust material, such as steel or plastic sheets, which can also handle long-term variable atmospheric conditions. Another important role of the external shell is to provide good sound isolation in order to keep the generated noise inside the acoustic enclosure. The framework of the acoustic cover can be either a part of the exterior or a standalone frame, especially in the case of mid- to large-size acoustic enclosures or more complex enclosure shapes.

Materials with high acoustic absorption coefficients are used for the internal surface of the enclosure. The sound pressure generated by the machinery inside the enclosure should be dissipated at the acoustic material in order to reduce the noise that can escape from the enclosure [6]. The application of poroelasitc materials for acoustic enclosures was investigated in [7]. Double-wall sandwich panel construction was found to be more effective than singe and perforated panels, especially in a low range of frequencies [8,9]. The vibration and noise generated during the operation of a machine is often transmitted or leaks into the foundations and other nearby objects, causing additional noise [6]. Therefore, in most cases, acoustic enclosures include passive sound and vibration isolation elements at the floor surface. Potential noise leakage can occur at service hatches and doors, elements necessary to inspect or service the machine enclosed in the acoustic housing. The active control of enclosure wall vibration has been investigated by many researchers [10,11]. In the case of semi-open enclosures or mufflers, acoustic material is placed on flow channels and diffusors to reduce the noise escaping from the enclosure.

**Figure 1:**
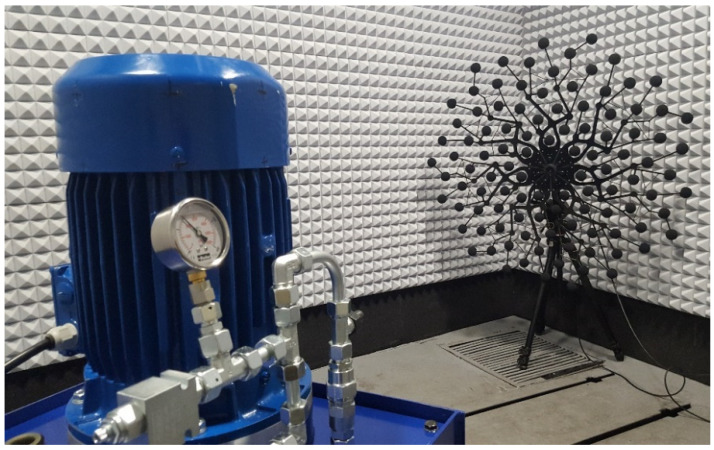
Hydraulic power pack placed in semi-anechoic chamber—noise source localization. Strong increase in temperature during experiments.

In our research in the field of noise in hydraulic systems presented in [12], including numerous acoustic experimental investigations on hydraulic power packs placed in a semi-anechoic chamber, a strong increase in both ambient and hydraulic fluid temperate was observed. In order to conduct the experiments at constant temperature conditions, pauses in between measurements with additional cooling and venting of test area were necessary (Figure 1). The same phenomenon of temperature increase was confirmed by end users of acoustic enclosures of hydraulic power packs.

### 1.2. Heat Accumulation

The efficiency of devices and systems used in engineering may wary strongly between objects and is defined by the type of machine, operation principle physical phenomena occurring during operation, etc. It can be unequivocally stated that some kinds of losses are always present in any kind of industrial setup. In most cases, the energy lost due to the limited efficiency of the device is transformed into heat and, to some smaller degree, into noise and vibration. Depending on the size and power of the considered system, the amount of generated heat can be significant. Automotive combustion engines are a great example of low-efficiency, small-size power units, with an efficiency ranging from 0.2 to 0.4. The amount of power that goes into heat in the case of the mentioned combustion engines can reach 60 to 70%. Another good example of heat generation due to a lack of efficiency can be found in fluid power systems. High power density coupled with efficiency ranging from 0.5 to 0.8 depending of the system size and type contributes to high temperatures generated during the operation of such systems. The accumulation of heat in the case of hydraulic systems is especially problematic due to the increased wear of the components at elevated temperatures of hydraulic medium, and the increased production of varnish and sludge [13,14,15,16]. Undoubtedly, many more examples of heat generation due to a lack of efficiency can be found in engineering. On the other hand, in some cases, the efficiency of a device itself may not cause high operation temperature. Some industrial devices transport or handle hot materials, such as fluids or parts, which cause higher operation temperatures.

The implementation of an acoustic enclosure to a low-efficiency or nominally exposed heat device that could dissipate the heat and operate under acceptable temperature conditions beforehand can now seriously limit heat transfer and cause higher operating temperatures. Engineering practice shows that the increase in temperature of most mineral-based hydraulic oils of 5 °C to above 60 °C will reduce the lifespan of such fluid to half of the nominal value [17]. The same negative effect of the increase in the operation temperature is present in the case of electric and electronic devices, where the mean time to failure is strongly influenced by the operation temperature [18].

Acoustic enclosures provide the isolation and absorption of noise, but will also ensure some thermal isolation, in many cases significantly increasing the operation temperature of enclosed devices. The obvious solution to the increased temperature due to enclosing in absorbing and insulating material is the application of additional cooling. Water heat exchangers are, in most cases, too expensive to implement due to their high costs of operation. Air coolers are used significantly more often in the case of acoustic enclosures. Forced air flow can effectively reduce the negative impact of acoustic enclosure; unfortunately, the application of fans and demand inlet and outlet air flow channels at the enclosure will compromise the effectiveness of the noise reduction when not properly designed and equipped with acoustic materials.

### 1.3. Heat Transfer Simulations

Computational fluid dynamics (CFD) is a technique widely used for fluid flow-related analyses and technical problems. It has been implemented successfully in many industrial and civil applications in the fields of aerodynamics, hydrodynamics, energy generation and energy transfer phenomena [19]. Referring to the previous sections of this paper, CFD was also successfully used to investigate and analyze heat transfer cases. Heating, Ventilation Air Conditioning (HVAC) applications of CFD have been presented in numerous studies. Some of them are based on component optimization, such as heat exchangers, compressors or fans [20,21,22,23]. The application of flow channel inserts and vortex generators and their influence on heat transfer, fluid flow and pressure have been investigated with the use of CFD by numerous researchers. The finite volume method was used to investigate several shapes of fin-plate heat exchanger vortex generators and their impact on the heat exchange performance [24]. Several vortex tube design parameters were investigated in regard to heat transfer and flow characteristics in [25]. The performance of twisted-type inserts in heat transfer and flow in nanofluid was investigated in [26].

The simulation of airflow inside a wind tower model including the heat transfer devices was presented in [27]. Thermal comfort inside an office equipped with an HVAC system was evaluated with the use of the finite volume method in [28]. The application of aerogel in building isolation was investigated by means of CFD simulations and experimental research in [29].

The finite volume method was also successfully applied in automotive engineering. For example, the fluid thermal environment around passenger car occupants was modeled, analyzed and redesigned with commercial CFD software packages [30]. Commercial truck underhood airflow-cooling factors were assessed and analyzed with the use of CFD modelling [31]. The problem of compromised heat exchange due to applied acoustic treatment was investigated in [32]. The application of acoustic panels near the thermally activated building system (TABS) caused a reduction in heat transfer, thus reducing TABS performance. CFD and experimental investigations were carried to identify the phenomena and compare the effects of horizontal and vertical sound absorbing solutions on heat exchange performance. The influence of acoustic enclosure on the natural convection of an air core reactor was investigated by means of a combined CFD and Taguchi method [33]. Several cases of acoustic cover with different main geometrical parameters were investigated in regard to natural convection performance optimization.

## 2. Numerical Modeling

Computational fluid dynamics can be used to simulate the change in the device operation temperature due to applied acoustic treatment in the form of an enclosure outfitted with forced air flow cooling. Numerical modeling of thermal phenomena occurring in the acoustic enclosure was conducted with the use of the FVM (finite volume method) in Ansys Fluent software. All calculations were performed in a three-dimensional domain in a steady state. In the simulations, the k-ε turbulence model was used.

The energy equation is described in the following equation (Equation (1)) [34]:

(1) $$\frac{\partial }{\partial t}\left(\varrho E\right)+\nabla \cdot \left(\vec{v}\left(\varrho E+p\right)\right)=\nabla \cdot \left(k_{eff}\nabla T-\underset{j}{\sum }h_{j}\vec{J_{j}}+\left(\overset{═}{\tau_{eff}}\cdot \vec{v}\right)\right)+S_{h}$$

The first two terms on the left-hand side of Equation (1) represent the unsteady term and the convection term, respectively, while the first three terms on the right-hand side of Equation (1) represent energy transfer due to conduction, species diffusion, and viscous dissipation, respectively. *S_h_* is an enthalpy source and includes volumetric heat sources defined in the analysis.

In Equation (1), the total energy *E* is defined as:

(2) $$E=h-\frac{p}{\rho }+\frac{v^{2}}{2}$$

where sensible enthalpy *h* for incompressible flows is described as:

(3) $$h=\underset{j}{\sum }Y_{j}h_{j}+\frac{p}{\rho }$$

In Equation (3), the species enthalpy *h_j_* is:

(4) $$h_{j}=\int_{T_{ref}}^{T}c_{p,j}dT$$

The value for *T_ref_* in the sensible enthalpy calculation for the pressure-based solver is 298.15 K.

The heat transfer *q* in the forced convection analysis is calculated from Newton’s law of cooling (Equation (5)):

(5) $$q=\bar{h}\left(T_{body}-T_{env}\right)=\bar{h}∆T$$

In general, the heat transfer coefficient *h* is not constant but is usually a function of the temperature gradient.

### 2.1. Geometrical Model

**Figure 2:**
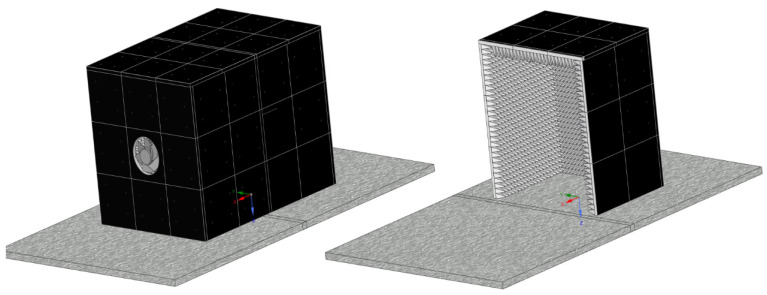
Acoustic enclosure example.

The acoustic enclosure presented in Figure 2 was chosen as the simulation object. The external shell of the enclosure consists of steel sheet metal. The inside of the enclosure was fitted with a layer of acoustic absorption material. The size of the enclosure is suitable for a semi-sized hydraulic power pack outputting 10 kW of power. It can be assumed that roughly one-third of this power will be transformed into heat at the fluid reservoir.

**Figure 3:**
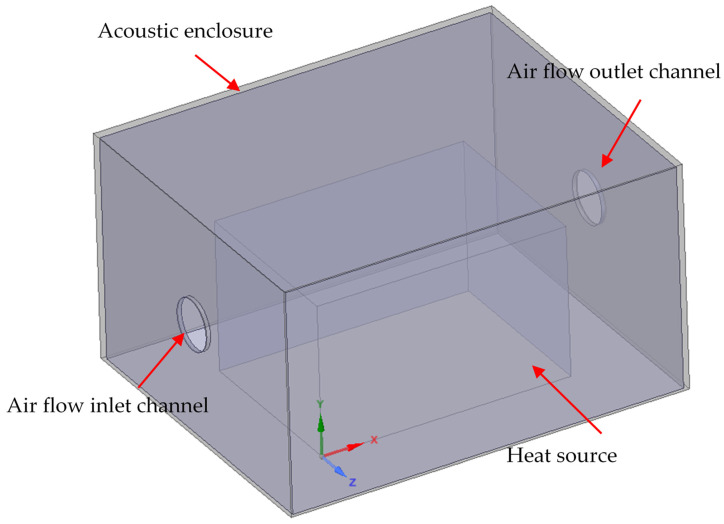
A geometrical model and a schematic view of boundary condition locations.

The geometry presented in Figure 2 was simplified to a 1300 × 1600 × 900 mm^3^ surface cuboid, as shown in Figure 3. The heat-emitting element with 3 kW of heating power was set into the enclosure and represented by another 700 × 1000 × 600 mm^3^ surface cuboid. The acoustic layer thickness was set to 15 mm, and the sheet metal outer shell was modeled as fully reflective. The fluid domain was defined by the air between the emitting source and the inside of the enclosure. The heat emission at the source was not modeled as a solid, instead a set of thermal conditions was applied on the source surfaces. The acoustic enclosure model also included a 200 mm air inlet and outlet channel, as shown in Figure 3.

### 2.2. Discrete Model

**Figure 4:**
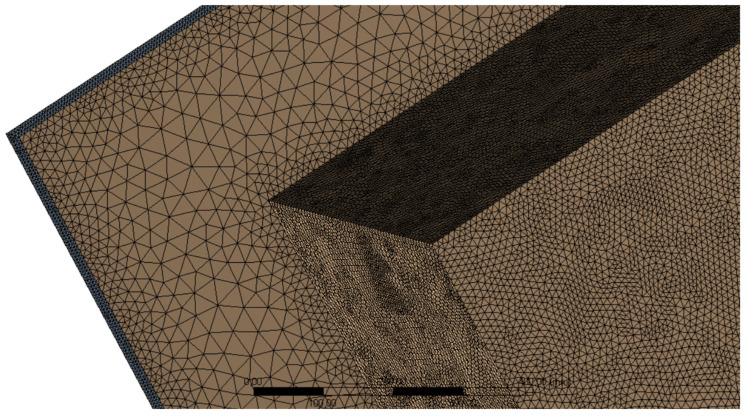
Discrete model—various mesh sizes at surfaces.

The finite volume mesh consisted of 10 million elements. The maximum element size in the fluid domain was set to 50 mm. At the interfaces of the heat-emitting source and fluid domain, as well as the acoustic enclosure and fluid domain, the mesh was refined to a maximum element size of 10 mm. The acoustic enclosure was discretized with the max element size of 5 mm. A partial view of the discretized model is presented in Figure 4.

### 2.3. Grid Sensitivity Check

The accuracy of the results was verified by the grid sensitivity test, which included different mesh refinements. The maximal mesh element size was changed in the air domain from 10 mm to 80 mm with the step of 10 mm, which provided 8 simulations in total.

**Figure 5:**
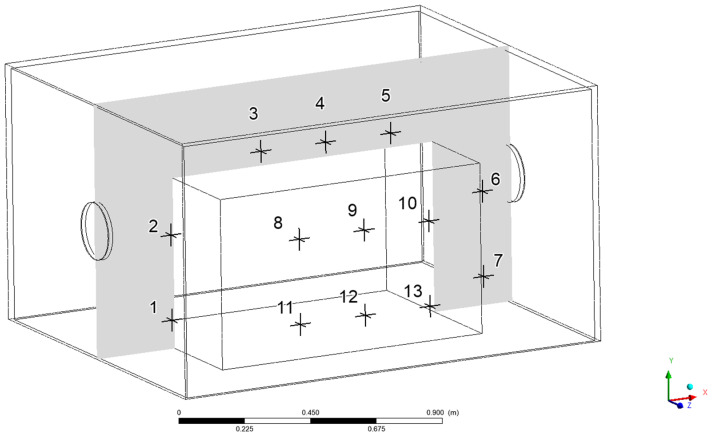
The location of temperature measurement points.

The inlet volume flow rate was 420 m^3^/h for each case. As the comparative value, the temperature was taken in the measurement points specified in Figure 5.

**Figure 6:**
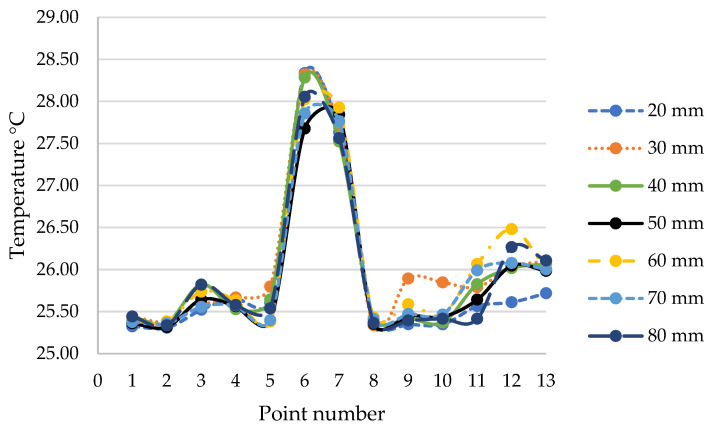
Grid sensitivity results.

The analysis results (Figure 6) showed that the temperature differences in particular points were small, the relative error did not exceed 0.007 °C and the maximum standard deviation was 0.792. Based on the results of the model sensitivity analysis, the simulations focused on the model having a maximum discrete element size of 50 mm, for which the calculated temperature values remained within the confidence interval. In Table 1, the detailed grid sensitivity results are compiled.

### 2.4. Material Properties and Boundary Conditions

The material properties of air, steel and acoustic material were chosen based on literature values. Table 2 presents the crucial material properties introduced to the numerical simulation.

The application of forced air flow through the acoustic enclosure should reduce the temperature increase. The assumption is made that the air temperature inside the enclosure should be maintained at 25 °C. The effectiveness of heat exchange will strongly depend on the air flow rate Q (m^3^/h), which is equal to the product of the amount of air exchange cycles per hour k (1/h) and the enclosure volume. Assuming the standard k value for machine rooms k = 30 and the volume of considered acoustic enclosure presented in Figure 3, the minimum fan air flow rate should be equal to 56 m^3^/h. In this study, a set of simulations was performed, including the inlet volumetric flow rates of 52.5 m^3^/h, 105 m^3^/h, 210 m^3^/h and 420 m^3^/h. The highest volumetric flow rate in a range of conducted research results from the analysis of commercially available industrial fans, and the specific nature of hydraulic power pack heat emission. It is important to note that the application of forced air flow as cooling can compromise the acoustic properties of enclosures. Additional acoustic measures in the form of flow channel dampers and diffusors should be applied with the installation of a fan. Another point of consideration should be the acoustic performance of the industrial fan itself. It is quite common especially for larger centrifugal fans to cause noise problems during operation [35,36].

**Figure 7:**
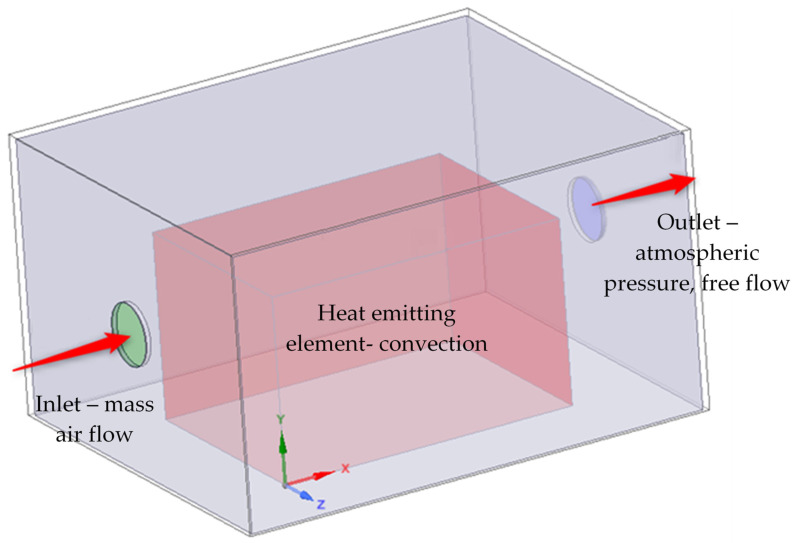
Computational fluid dynamics—boundary conditions.

The inlet channel boundary condition was set as the mass air flow of *Qm* and temperature of 25 °C. The outlet air channel boundary condition was set to atmospheric pressure *P* and free flow (Figure 7). The heat-emitting surfaces representing the generation of heat in the hydraulic power pack were set to a temperature of 40 °C and a heat transfer coefficient equal to 12$\frac{W}{m^{2}\cdot K}$ [37]. On the inner and outer walls of the enclosure, the heat flux boundary condition was set. The interfaces between the acoustic material and air were set to the coupled wall condition.

## 3. Simulation Results

**Figure 8:**
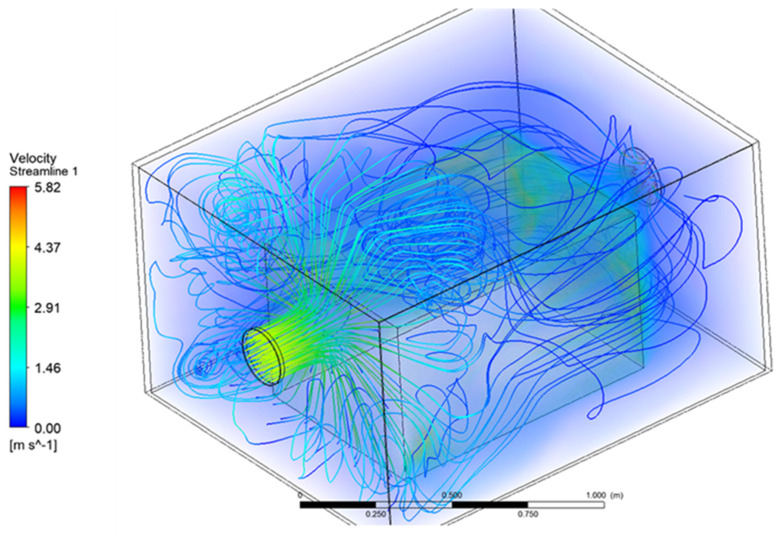
Three-dimensional air flow velocity current lines inside the acoustic enclosure.

**Figure 9:**
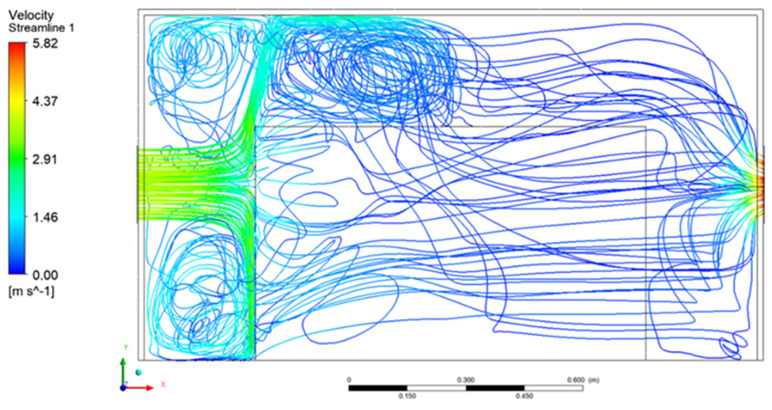
Air flow velocity current lines inside the acoustic enclosure—plane view.

The figures presented below show the graphical results obtained during simulations of 420 m^3^/h inlet flow rate. Air flow current lines inside the enclosure are presented in Figure 8 and Figure 9. The air flowed into the enclosure directly onto the frontal surface of the heat-emitting object where a strong current deflection took place. The air flow currents above the first part of the heat source showed turbulences, which should have had a positive effect on the heat removal from the acoustic enclosure.

**Figure 10:**
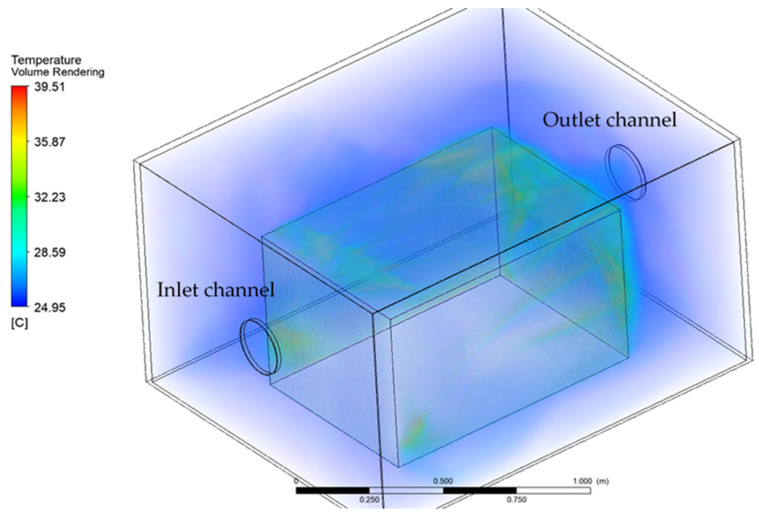
Temperature distribution in the simulation domain—inside of the acoustic enclosure.

**Figure 11:**
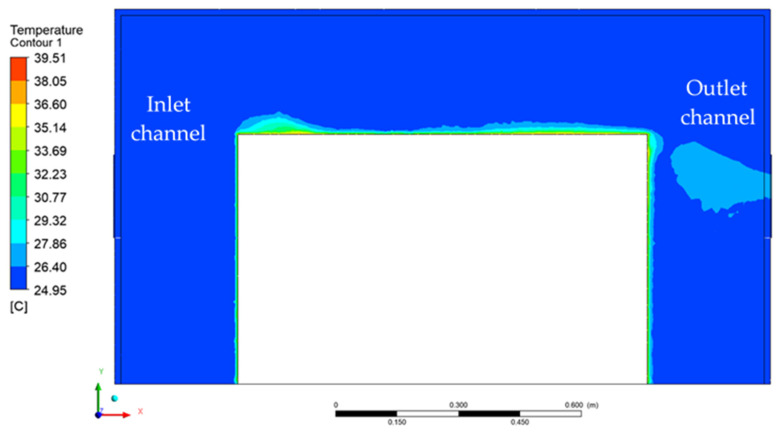
Temperature distribution on the YX acoustic enclosure symmetry surface.

The temperature distribution inside the acoustic enclosure computed in simulations is presented in Figure 10. The heat emitted from the source was convected by the air and was removed from the enclosure. Some areas of fluid around the heat source exhibited higher temperature values. This varied distribution of temperature at the source surface was caused by the voids in air flow velocity created by the turbulences. It could be observed that the fluid temperature increased in direct proximity to the heat source, but due to forced air flow, the average temperature inside the enclosure did not change significantly. Figure 11 presents the temperature distribution on the symmetry plane of the model. A veil-shaped area of increased temperature could be observed at the outlet channel of the enclosure, which represents the heat removal.

**Figure 12:**
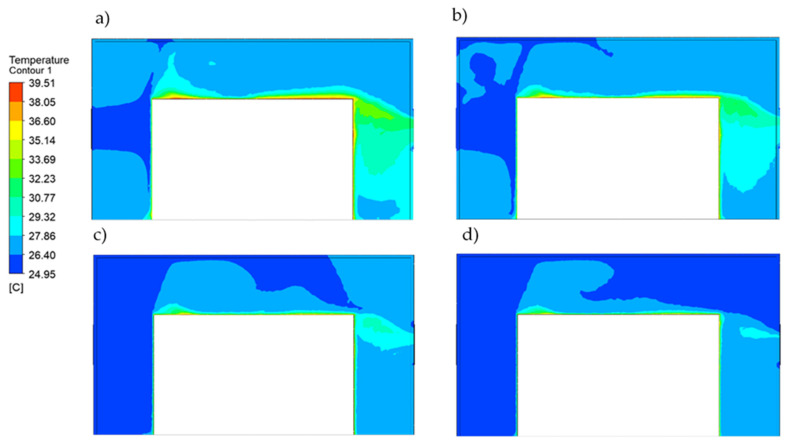
Temperature distribution on the YX acoustic enclosure symmetry surface for the flow rates of: (**a**) 52.5 m^3^/h, (**b**) 105 m^3^/h, (**c**) 210 m^3^/h, (**d**) 420 m^3^/h.

In order to check the impact of the flow rate on the temperature distribution in the enclosure, the additional simulation results with flow rates of 52.5 m^3^/h, 105 m^3^/h and 210 m^3^/h are presented in Figure 12. The plots present the temperature distribution at the XY symmetry plane of the model for each analyzed case. The minimum temperature was 24 °C, and the maximum temperature was equal to 40 °C. It can be seen that the much smaller flow rates of 52.5 m^3^/h and 105 m^3^/h did not provide sufficient cooling of the hydraulic power unit, and the temperature in the majority of the enclosure volume reached almost 30 °C.

**Figure 13:**
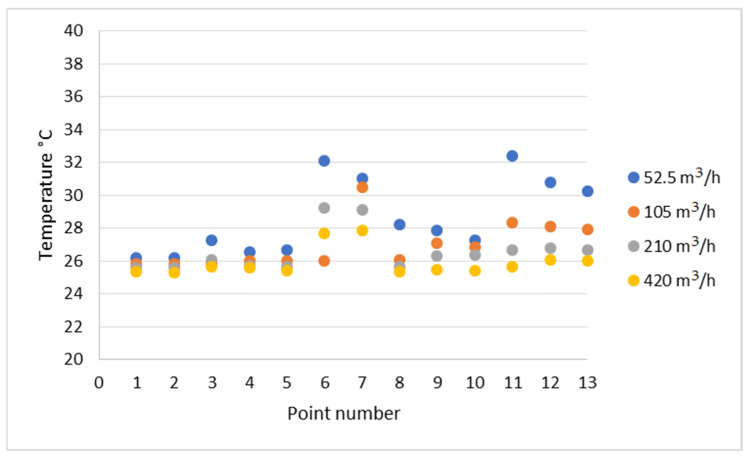
The plot of temperature values in specified measurement points for the flow rates of: 52.5 m^3^/h, 105 m^3^/h, 210 m^3^/h and 420 m^3^/h.

For the selected points inside the enclosure, the precise temperature values were checked. Due to the symmetricity of the model, further analysis of the temperature distribution points was carried out mainly in the frontal half of the fluid domain volume. The location of the measurement points is presented in Figure 5. Based on the results shown in Figure 13, the previous conclusion about the cooling effectiveness of higher volume flow rates of 210 m^3^/h and 420 m^3^/h is confirmed.

## 4. Conclusions

The conducted computational fluid dynamics calculations showed that acoustic enclosures equipped with proper forced air flow do not cause heat buildup inside the enclosure at the considered operating conditions. The right air flow rate generated by applied fans can induce air exchange in the enclosure volume frequently enough to prevent an increase in operating temperature. The analysis of the temperature values in the selected points of the enclosure interior (Figure 13) showed that for the frontal (points 1 and 2) and side surfaces (points 8, 9, 10) of the hydraulic unit at the height of the air inlet channel, the smaller flow rates of 210 (m^3^/h) and 105 m^3^/h provided good thermal energy removal. However, the rear surface (points 6 and 7) and side surfaces at the lower areas of the hydraulic unit (points 11, 12, 13) were efficiently cooled only at the flow rate of 420 m^3^/h.

The outcomes of this study provide a useful insight into the heat exchange phenomena in acoustic enclosures needed to prepare and design experimental investigations in the future research. The presented numerical model can be applied to other acoustic enclosures, with modified boundary conditions, such as flow rates, volumes, ambient temperature, type of enclosed device and the material properties of both enclosure and heat generation objects. The simulation approach presented in this paper can also be applied for the optimization of air flow rates and effective fan size, at an allowed temperature increase inside the enclosure.

An important consideration might appear in the case of higher power heat sources. Air flow rates necessary for effective cooling might reach larger values, thus forcing the use of larger more powerful industrial fans, which often contribute strongly to the overall machine sound power level. The influence of restrictions in air flow channels, such as ducts and mufflers, which might be present in an acoustic enclosure should also be considered in computational analysis, especially at higher air flow rates and more powerful heat sources.

## Figures and Tables

**Table 1:** Grid sensitivity results.

Measurement Point	Temperature Mean Value °C	Rel. Error	Stand. Dev.
1	25.37	4.63 $\times 10^{-4}$	$7.61\times 10^{-2}$
2	25.37	$1.98\times 10^{-3}$	$5.43\times 10^{-2}$
3	25.69	$1.70\times 10^{-3}$	$1.27\times 10^{-1}$
4	25.63	$2.55\times 10^{-3}$	$9.40\times 10^{-2}$
5	25.54	$5.78\times 10^{-3}$	$1.47\times 10^{-1}$
6	27.81	$4.85\times 10^{-3}$	$7.92\times 10^{-1}$
7	27.64	$7.38\times 10^{-3}$	$2.34\times 10^{-1}$
8	25.44	$3.20\times 10^{-3}$	$1.49\times 10^{-1}$
9	25.53	$3.12\times 10^{-3}$	$1.81\times 10^{-1}$
10	25.50	$2.32\times 10^{-3}$	$1.66\times 10^{-1}$
11	25.83	$7.24\times 10^{-3}$	$2.97\times 10^{-1}$
12	26.00	$1.60\times 10^{-3}$	$3.19\times 10^{-1}$
13	25.92	$2.44\times 10^{-3}$	$2.49\times 10^{-1}$

**Table 2:** Material parameters defined in CFD simulation.

Material	Density kg/m^3^	Specific Heat J/(kg·K)	Thermal Conductivity W/(m·K)
Air	1.225	1006.43	0.0242
Acoustic material	35	2100	0.035
Steel	8030	502.48	16.27

## Data Availability

The data presented in this study are available on request from the corresponding author.

## Nomenclature

E	total energy (J)
h_j_	species enthalpy (J)
$\bar{h}$	average heat transfer coefficient (W/m^2^K)
J_j_	mass flux (kg/m^2^s)
$\vec{J_{j}}$	the diffusion flux of species j
K	thermal conductivity (W/mK)
k_eff_	effective conductivity, k_eff_ = k + k_t_
k_t_	turbulent thermal conductivity, defined according to the turbulence model being used
p	pressure (Pa)
t	time (s)
T	temperature (K)
T_body_	temperature of object’s surface (K)
T_env_	temperature of the environment (K)
q	heat transfer (W, J/s)
v	velocity magnitude (m/s)
$\vec{v}$	overall velocity vector (m/s)
Y_j_	mass fraction (-)
ρ	density (kg/m^3^)
$\overset{═}{\tau_{eff}}$	stress tensor (Pa)
